# Aquatic versus terrestrial attachment: Water makes a difference

**DOI:** 10.3762/bjnano.5.252

**Published:** 2014-12-17

**Authors:** Petra Ditsche, Adam P Summers

**Affiliations:** 1 Friday Harbor Laboratories, University of Washington, 620 University Road, Friday Harbor, WA, 98250, USA

**Keywords:** adhesion, biofilm, friction, hooks, suction

## Abstract

Animal attachment to a substrate is very different in terrestrial and aquatic environments. We discuss variations in both the forces acting to detach animals and forces of attachment. While in a terrestrial environment gravity is commonly understood as the most important detachment force, under submerged conditions gravity is nearly balanced out by buoyancy and therefore matters little. In contrast, flow forces such as drag and lift are of higher importance in an aquatic environment. Depending on the flow conditions, flow forces can reach much higher values than gravity and vary in magnitude and direction. For many of the attachment mechanisms (adhesion including glue, friction, suction and mechanical principles such as hook, lock, clamp and spacer) significant differences have to be considered under water. For example, the main principles of dry adhesion, van der Waals forces and chemical bonding, which make a gecko stick to the ceiling, are weak under submerged conditions. Capillary forces are very important for wet adhesion, e.g., in terrestrial beetles or flies, but usually do not occur under water. Viscous forces are likely an important contributor to adhesion under water in some mobile animals such as torrent frogs and mayflies, but there are still many open questions to be answered. Glue is the dominant attachment mechanism of sessile aquatic animals and the aquatic realm presents many challenges to this mode of attachment. Viscous forces and the lack of surface tension under submerged conditions also affect frictional interactions in the aquatic environment. Moreover, the limitation of suction to the pressure difference at vacuum conditions can be ameliorated under water, due to the increasing pressure with water depth.

## Introduction

Attachment in animals, plants and microorganisms serves a variety of functions: the interconnection of body parts, fixation of the whole animal, a locomotor structure, or eggs to the substrate, and forming a stable platform for copulation, feeding, phoresy, parasitism or predation [1–2]. Here we focus on attachment of animals to stiff, solid substrates under two biologically relevant conditions – in a dry or humid environment (terrestrial) in comparison to completely submerged in water (aquatic). Our aim is to provide a framework for considering these two conditions of attachment. We will contrast the forces that act to dislodge an attached organism in submerged and emerged environments and distinguish between the attachment forces in these disparate arenas. The difference between a submerged attachment event and one that is dry is neither clear-cut nor simple. Consider the classic example of a toe of a gecko adhered to a leaf. On its face, this is a case of terrestrial adhesion, but depending on humidity there could be a monolayer of water on the surface of the leaf, or there might be a patch of standing water on the leaf after rainfall. Even in this terrestrial example, there is the possibility of submerged mechanics applying at some size scale. Similarly, there are cases in the fully submerged environment where terrestrial mechanics might apply. For example, when spiders bring with them a ball of air as they dive beneath the surface, or when two superhydrophobic surfaces interact underwater. Therefore, the first task that we face is to make clear what we mean as we try to distinguish between these two environments, while also keeping in mind that there is a continuum and counterintuitive exceptions to the framework we are proposing.

For our purposes, a terrestrial environment has air as surrounding fluid. However, the air can be completely dry or rather humid. Therefore, effects of water can play some role in a terrestrial environment, especially in form of capillary forces. The aquatic or immersed environment is one in which water surrounds the organism completely, or at least the entire attachment organ and the attachment surface. Here, water plays a central role and must be considered to be surrounding and separating the two surfaces brought into contact. Some unusual immersed attachment examples might be an insect stepping into a droplet of water sitting on a branch. The size scale of the droplet is such that the entire attachment process is occurring underwater. However, in this very example we can see a gray area in that the foot has recently been dry, so the tendency of air to surround the attachment organ as it penetrates the droplet may be important. Our generalizations about aquatic environments apply when the foot of the insect brings none of the terrestrial environment with it into the aquatic environment.

The natural world is replete with examples of aquatic attachment and terrestrial attachment in the sense that we propose. In the terrestrial realm, virtually every case of arthropod attachment, from flies on ceilings to a spider dancing on a web is an example. There are examples in diverse taxa, including suctorial bats, several variations of lizards, and countless beetles, spiders and ants. In every stream, there are mobile larvae that spend their lives attaching to the substrate. These insects are ruled by the water forces imposed by local flow conditions. There are vertebrate examples in waterfall climbing gobies and frogs, and echinoderms that adhere in the intertidal zone. In short, though there is certainly a continuum between aquatic and terrestrial conditions, the vast majority of biological adhesion takes place in a system dominated by one extreme or the other.

## Review

### Forces that act to dislodge

**Figure 1:**
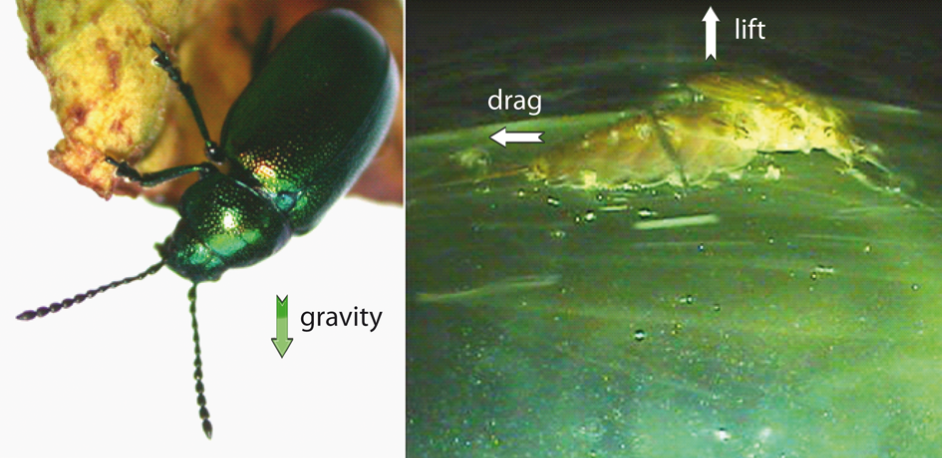
A) Green dock beetle *Gastrophysa viridula* as an example for terrestrial attachment (image: Stanislav Gorb, reproduced with permission by the author), B) Mayfly larvae *Epeorus* attaching to a substrate in running water (image: Petra Ditsche). Arrows show the direction of the most important forces acting on the animals.

Attachment to the substrate is aimed at either locomotion or staying in place [3]. For either purpose, the animal has to overcome forces acting to dislodge it, and it is in the nature of these forces that terrestrial and aquatic systems vary widely. The forces are not the same in terrestrial systems compared with aquatic ones in either scale or type (Table 1). While in terrestrial systems gravitation is the most relevant, in aquatic systems gravity is mostly cancelled by buoyancy. In contrast, in aquatic systems flow forces such as drag and lift are very important while they are seldom substantial in terrestrial systems. The magnitude of these forces varies by environment and so does the direction: while gravity always acts only in the direction of the earth, drag pushes the animal across the surface, and lift can pull it off regardless of orientation (Figure 1). Moreover, aquatic flows are often variable in magnitude and direction on very short time scales. In running waters and some directed marine currents there is a general main flow direction, while the waves in the marine intertidal move the water in different directions with high frequency. In many cases, water flow is not laminar (laminar means particles do not cross streamlines) but rather turbulent, with sudden, stochastically determined changes in direction [4]. There are also important issues of scaling to consider that some detachment forces will scale with the cube of animal length while others scale with the square.

**Table 1:** Forces of detachment in terrestrial and aquatic environments. Relative importance: 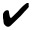 – usually important, 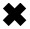 – usually not important.

	terrestrial	aquatic	direction	magnitude	determining parameters

gravity	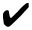	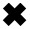	predictable	predictable	mass, acceleration of gravity
buoyancy	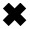	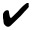	predictable	predictable	volume, density
inertial forces	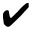	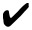	unpredictable	unpredictable	mass, acceleration
lift and drag forces, acceleration reaction force	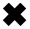	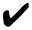	unpredictable	unpredictable	shape and size, orientation, velocity, fluid density and viscosity

In both the terrestrial and the aquatic environment the attachment event is surrounded by fluid, in one case air and in the other water. Air and water have different properties that have several consequences for the organisms living in these fluids [5]. Three principle factors have a significant effect on the types of forces acting to dislodge attached organisms. The first is density; water is around 1000 times denser than air, which means that both inertia and buoyancy are very different in these two fluids (Table 2). Second, air has just around 1.8% of the (dynamic) viscosity of water, which affects the scale of forces between the fluid and the organism. Thirdly, water is a polar liquid while air is a largely inert gas. This difference has profound effects on physicochemical interactions in the dry and in the wet environment.

**Table 2:** Density and viscosities of air and water at 20 °C.

parameter	unit	air	freshwater	saltwater

density	[kg/m^3^]	1.205	0.998 × 10^3^	1.024 × 10^3^
dynamic viscosity	[Pa·s]	18.08 × 10^−6^	1.002 × 10^−3^	1.072 × 10^−3^
kinematic viscosity	[m^2^/s]	15.00 × 10^−6^	1.004 × 10^−6^	1.047 × 10^−6^

#### Gravity

Gravity is usually the most important force acting on the attachment of terrestrial animals. The gravitational force on an organism is simply

[1]
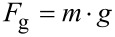


and always acts in the direction of the centre of the Earth (*g*: gravitational acceleration). The magnitude of the gravitational force (*F*_g_) scales with the mass of the organism (*m*) and therefore with the cube of any length parameter. The magnitude of the gravitational force is the same in terrestrial and aquatic environments and, for biological purposes, it is constant regardless of time or place. Gravity is the archetype for a predictable force, though the frame of reference of the organism does not always point in the ventral direction. In Figure 1, the beetle is attached to a substrate above it and the gravitational vector points almost exactly opposite to the attachment force.

#### Buoyancy

The density of water (ρ_w_) is much higher than the density of air and is closer to the typical density of living organisms (ρ_a_), so buoyancy can substantially offset the gravitational force in the aquatic environment whereas in the terrestrial environment it is usually negligible. The buoyant force (*F*_b_) always acts in the opposite direction to the gravitational force and is defined as

[2]
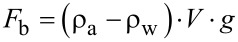


Buoyancy, like gravitational force scales with the cube of a length parameter because it scales with the displaced volume (*V*). It varies little with time and in space as neither density nor volume typically vary rapidly. There are interesting exceptions to this rule of thumb that have implications for attachment. Organisms with an air compartment in an aquatic environment must deal with changes in volume imposed by the ideal gas law. The volume of the compartment will vary locally with pressure and temperature. For organisms able to transit the first 10 m of depth in an aquatic environment, this is a 50% decrease in volume when descending or a doubling when ascending. This implies a similar change in the buoyant force while the offsetting gravitational force would remain the same. Under some conditions, it is possible to have the buoyant force exceed gravity. This positive buoyancy can require an organism to attach to an underwater substrate just to keep from floating to the surface.

#### Inertial forces

For an animal sitting on a leaf moving in the wind, an insect landing on a substrate, a clingfish attaching to kelp moving in the current, or simply during walking on a substrate, inertial forces contribute to detachment. Whether the surface or the organism is in motion, there are forces associated with changes in velocity

[3]
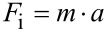


The direction of inertial forces (*F*_i_) is determined by direction of the acceleration vector, and the magnitude is dependent on both mass (*m*) and acceleration (*a*). The dependence on the mass implies a scaling coefficient of the cube of a length parameter, but the picture is complicated by the nature of the scaling of acceleration. Although the rules for the scaling of acceleration are not well-described, in many systems there is an inverse scaling such that very high accelerations are found in very small systems. There is no equation for the scaling of inertial forces that takes into account both acceleration and mass (although see [6] for some special cases that are well-described). Neither the direction nor the magnitude of inertial forces is affected by water, so they are the same in both in aquatic and terrestrial environment. The predictability of inertial forces is linked to the predictability of acceleration and it is difficult to make a case that this will vary with environment.

#### Flow forces

In aquatic systems drag and lift forces are the most important forces acting on an attached organism [4,7–8]. Drag and lift have several causes and depend on a variety of parameters. They are both highly variable in magnitude and direction and, thus, hard to predict. Drag is a force due to fluid movement that acts in the direction of the free stream flow. It has two components, friction drag (or skin friction) and pressure drag (or form drag), which both depend on shape and fluid parameters but in quite different ways [4]. Friction drag is caused by friction of the water flowing over the surface of the animal body. It varies directly with the viscosity of the fluid [4] and the wetted surface area of the organism. Pressure drag is caused by the wake formed downstream of an animal. This turbulent wake creates a low pressure zone downstream of the animal [9]. Friction drag is proportional to the product of surface area and velocity, while pressure drag is proportional to the product of the area in frontal projection and velocity squared. Pressure drag (*F*_d_) can be calculated by the drag coefficient (*C*_d_), the density of the fluid (ρ), the area in frontal protection (*S*_f_) and the flow velocity (U):

[4]
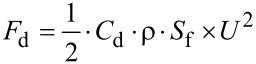


In the same medium the importance of these two types of drag varies with flow speed and the size of the animal. Friction drag dominates for small animals and slow flow speeds, and pressure drag dominates for large animals and/or fast flow speeds. In either regime, the resistance of an animal to the water flow is determined by its body shape. Of course, animals of the same body shape and size will experience much higher drag forces at the same flow velocity in water compared to air (see below in Table 3), because of the higher density and viscosity in water.

Lift, acting at right angles to the free stream of flow, is dependent on the shape and is proportional to the projected planform area (*S*_p_) of the organism, the fluid density and the square of the fluid velocity:

[5]
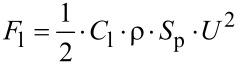


The dependency on *U*^2^ means that the lift force as well as drag force are very important components of the total detachment force. The lift coefficient (*C*_l_) and the drag coefficient describe the effect of shape.

In unsteady flow another force matters, namely the acceleration reaction force [4]. Consider an animal accelerating during swimming, some water must move as well, so the animal not only accelerates its own mass but also this “added mass of the fluid” [10]. The same force occurs when water accelerates over a stationary object. The acceleration reaction force acting on an attached animal in a wave is proportional to its volume (*V*_a_) and the water acceleration [11]. It can be calculated from the added mass coefficient (*C*_a_), the density of the fluid, the volume of the animal and the acceleration.

[6]
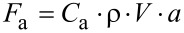


While drag is highest at the maximum velocity, the acceleration reaction force has its maximum when the acceleration is highest. For larger organisms the acceleration reaction force can be several times higher than drag force, while for small organisms drag matters most [11].

For example, let us consider a hemispherical attached organism, closely adhered to the substrate, in a terrestrial and an aquatic context. We will assume a mass of 2.2 g, a radius of 1 cm, an ambient temperature of 20 °C and flow velocities between 1 and 30 m/s, and for the purposes of a comparison of inertia we assign an acceleration of three times of Earth’s gravity [12–13]. From the literature we find that a hemisphere has a drag coefficient of 0.32 and a lift coefficient of about 0.75 [4,14]. Table 3 shows that in the terrestrial case the gravitational force is dominant, while in the aquatic system lift and drag matter. We can calculate that the density of the organism is 2.2 g/(4π/3·1 cm^3^)/2 = 1.05 g/cm^3^. The projected frontal area is *S*_f_ = (π·1 cm^2^)/2 =1.57 cm^2^, the planform area *S*_p_ = π·1 cm^2^ = 3.14 cm^2^ and the surface area is (4π·1 cm^2^)/2 = 12.57 cm^2^. With these parameters, we consider the relative magnitude of the detachment forces, bearing in mind that they may not act normal to the substrate. The force due to gravity is exactly the same in aquatic and terrestrial environments, but the net force when buoyancy is subtracted leads to a 20-fold higher force in terrestrial environments. This could release a constraint on size in the aquatic environment. Drag and lift, whether in relatively calm or extremely swift flows, are three orders of magnitude higher in the aquatic environment than on land. This suggests that shape might be far more variable in the terrestrial environment because there is less selective pressure to streamline. Moreover, drag and lift forces in aquatic environment can reach much higher values compared with gravity on land. Lastly, consider the inertial forces that are predicted on the basis of an plausible natural acceleration. They are the same in both environments, but they are three times higher than the static gravitational forces and 60 times higher than the net gravitational/buoyancy force for an aquatic organism. This brings home the importance of considering the movement of the attached organism. Accelerations due to locomotion or due to movement of the substrate may be a dominant force driving detachment. Furthermore, when considering safety factors expressed as a multiple of body mass, bear in mind that the inertial forces could easily exceed this safety factor three- or four-fold.

**Table 3:** Calculation of the detachment forces of a theoretical hemispherical organism (mass: 2.2 g and radius: 1 cm).

	forces in terrestrial environment [mN]	forces in aquatic environment [mN]

weight (gravitational force)	21.582	21.582
buoyancy	0.025	20.594
**weight − buoyancy**	**21.557**	**0.988**
drag (*U* = 0.5 m/s)	0.008	6.299
drag (*U* = 1 m/s)	0.030	25.195
drag (*U* = 2 m/s)	0.121	100.781
drag (*U* = 5 m/s)	0.757 (wind)	629.875
drag (*U* = 30 m/s)	27.270 (whole gale)	22.675 × 10^3^ (base of a waterfall)
lift (*U* = 0.5 m/s)	0.035	29.525
lift (*U* = 1 m/s)	0.141	118.103
lift (*U* = 2 m/s)	0.567	472.413
lift (*U* = 5 m/s)	3.547	2.952 × 10^3^
lift (*U* = 30 m/s)	127.700 (whole gale)	106.29 × 10^3^ (base of waterfall)
inertial force (*a* = 3*g*)	64.746	64.746

### Forces of attachment

Attachment mechanisms are diverse and we can categorize them into three types by the time course of operation: permanent, temporary and transitory [15–16]. Animals attaching themselves permanently to the ground for their whole (adult) lifetime are called sessile. Blue mussels or barnacles are very prominent examples of this sessile type. Other animals such as molluscs, claw-bearing aquatic arthropods or sea stars, use alternating attachment for locomotion or for short-time fixation (temporary attachment) and are called motile or mobile. An intermediate form between temporary and permanent attachment can be found, for example, in many marine larvae, allowing them to explore possible substrates prior to permanent attachment. Transitory adhesion allows simultaneous adhesion and locomotion on a viscous film as practiced by molluscs or some flatworms [16]. The boundary between temporary and transitory adhesion is not always clear [16].

Taking into account (1) the fundamental physical mechanisms, (2) the biological function and (3) the duration of attachment time, the attachment mechanisms have been divided into eight main functional principles: wet and dry adhesion, friction, suction and the mechanical principles hooks, clamps, locks and spacers [2]. We will discuss the differences between these fundamental principles when applied under water. In nature some animals combine these fixation principles, for example limpets use suction and glue, black fly larvae support their hooks by secretion and some squids combine suckers with hooks.

**Figure 2:**
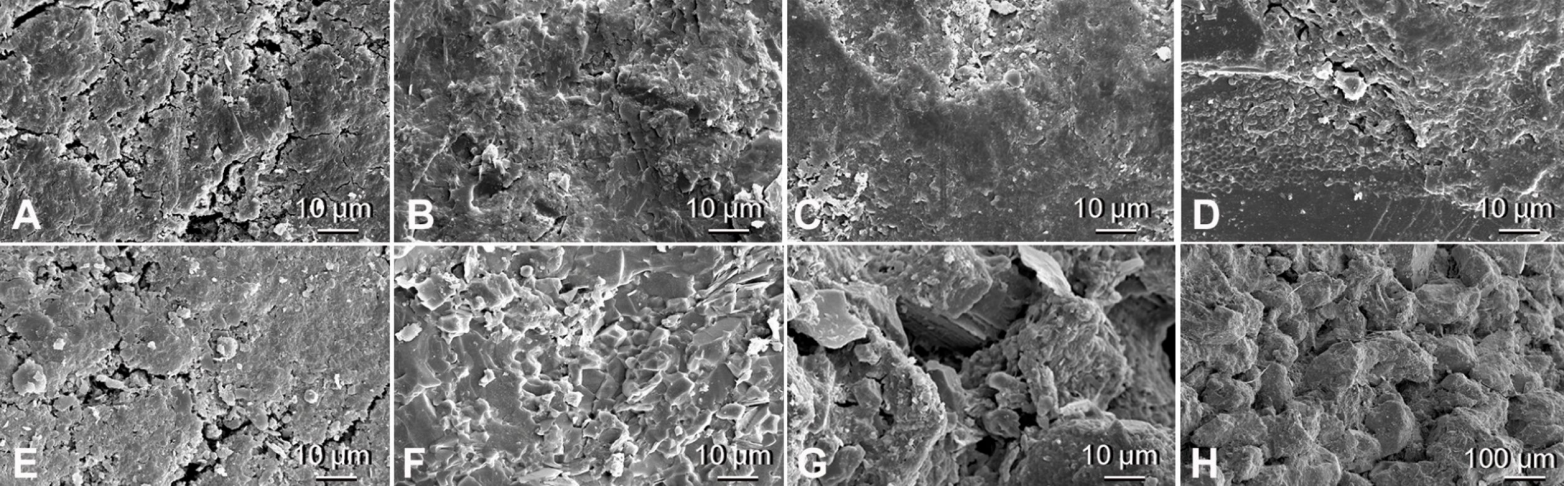
SEM pictures of the surface of selected rocks found in running waters: (A) andesite, (B) slate, (C) basalt, (D) quartz gravel (E) greywacke, (F) quartzite and (G, H) sand stone. (Reproduced from [18]).

Attachment is a two-body problem so there is interplay with the substrate, and the properties of the substrate also must be taken into account [17]. Common solid substrates in aquatic systems are stones, plants, wood pieces, artificial substrates and even other animals. Stones (Figure 2), artificial substrates and plants have large variation in surface texture, from smooth to very rough and smooth to hairy or covered with waxes [18–19]. Also the surface energy and with it the wettability of surfaces as well as the elasticity of the substrates are important properties, which can influence attachment [17].

**Figure 3:**
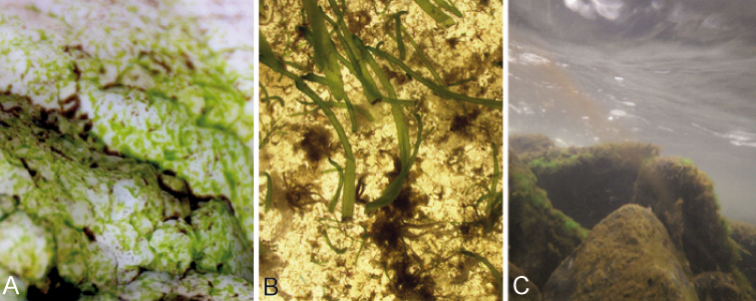
Examples of substrates which have been exposed to an aquatic environment and are covered with biofilm and periphyton; A) stone of the marine intertidal covered with biofilm and algae (image: Petra Ditsche); B) artificial substrates after 6 week exposure in the sea, with biofilm and growth of first macroalgae (image: Adam Summers); C) biofilm and macroalgae cover the stones of this stream in a thick layer (image: Sarah Kaehlert).

Another important point needs to be considered for aquatic systems. While most terrestrial animals make contact directly with the substrate, in aquatic environments the substrates are usually covered with a biofilm and fouling organisms (Figure 3). Biofilms play not only an important role in stimulating or inhibiting the settlement of fouling invertebrates [20–25], but they also change the surface properties of the primary substrate considerably and by this can affect the attachment forces significantly [26–27]. While biofilms can vary greatly in composition and thickness, they are usually softer than the primary substrate, and change the surface topography [26]. Moreover, microorganisms can change the wettability of the substrates surface, which is probably the reason for a different response of some larvae to these surfaces [28].

Some examples of attachment forces for different animals and attachment devices are given in Table 4 and Table 5. Very large attachment forces are generated by glue adhesion and suction. However, as the given values have been determined under very different conditions (substrates of different material, surface energy, roughness and elasticity; different measurement methods) the values are hard to compare and principally serve to demonstrate that wide-scale comparative studies are sorely needed in this field. Scale issues are also important; some studies are on a particular body part, e.g., the attachment of a single seta, while others are on whole organisms. This is certain to add substantial variability and we cannot calculate a reasonable attachment force for the whole animal from measurements of a single seta, tube foot or sucker.

**Table 4:** Examples for attachment parameters measured parallel to the substrate according to different authors (whole animal, on smooth substrates at water level and terrestrial environment, respectively).

taxon	attachment device	substrate	attachment force [N]	tenacity [kPa]	force/body mass	ref.

*Gekko gecko* (Gekkonidae, Reptilia)	adhesive pad (dry)	glass	17		≈17	[29]
		PMMA	27		≈27	
*Gekko gecko* (Gekkonidae, Reptilia)	adhesive pad (submerged)	glass	5		≈5	[29]
		PMMA	24		≈24	
*Staurois guttatus* (Ranidae, Amphibia)	adhesive pads (dry)	polyethylene	1165 (whole animal)	3.0 (pads)	43	[30]
*Staurois guttatus* (Ranida, Amphibia)	adhesive pads (submerged)	polyethylene	42 (whole animal)	0.1 (pads)	1.5	[30]
*Chthamalus fragilis* (Cirripedia, Crustcea) (ca. 8 mg)	glue	polystyrene	0.11	105		[31]
*Patella vulgata* (Patellidae, Gastropoda)	glue, sucker	glass	50	50		[32]
*Simulium vittatum* (Simuliidae, Insecta) (ca. 6 mm long)	circlet of hooks, secretion	wire	0.012			[33]

**Table 5:** Examples for attachment parameters measured perpendicular to the substrate according to different authors (whole animal, on smooth substrates at water level and terrestrial environment, respectively).

taxon	attachment device	substrate	attachment force [N]	tenacity [kPa]	force/body mass	ref.

*Staurois guttatus* (Ranidae, Amphibia)	adhesive pads (dry)	polyethylene	0.373 (whole animal)	2.4 (pads)	13.8	[30]
*Staurois guttatus* (Ranidae, Amphibia)	adhesive pads (submerged)	polyethylene	0.089 (whole animal)	0.1 (pads)	3.3	[30]
Bivalvia (species non def.)	glue	glass		320–750		[34]
*Patella vulgata* (Patellidae, Gastropoda)	glue, sucker	glass	up to 240	up to 230		[32]
*Lottia gigantea* (Patellidae, Gastropoda)	glue, sucker	Lucite		50		[35]
*Hapalothrix lugubris* (Blephariceridae, Insecta)	sucker	rock	0.084 (one sucker)			[36]
*Gobiesox maeandricus*, *Gobiesocidae*, *Actinpterygii/Pisces*	sucker	epoxy resin	up to 50	20–50	80–250	[37]

### Adhesion

A variety of different mechanisms contributes to adhesion: (i) mechanical interlocking on a very small scale, (ii) electrostatic forces, (iii) diffusion, (iv) chemical bonding as ionic, covalent or hydrogen bonds, and (v) dispersive or van der Waals forces. While the first three mechanisms of adhesion presumably contribute just a minor part to general adhesion, the latter two are generally accepted as the primary mechanisms in many systems [19]. The mechanisms involved in wet and dry adhesion are different (Table 6). Dry adhesion occurs in a dry environment and no fluid film is involved. When adhesion takes place in a humid environment, there is a substantial increase in adhesive forces [38]. Moreover, some animals secrete a liquid themselves [19,39]. If a fluid film is present, we have the conditions of wet adhesion. In wet adhesion two other forces contribute considerably to adhesion: (vi) capillary forces, and (vii) viscous forces. The latter is often called Stefan adhesion. A special case of wet adhesion is the secretion of adhesives (glue), which we will discuss below.

**Table 6:** The mechanisms of adhesion under dry, wet and immersed conditions. Relative importance: 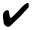 – usually important, 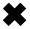 – usually not important.

	dry conditions	wet conditions (liquid film)	immersed conditions

mechanical interlocking	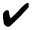	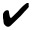	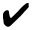
electrostatic forces	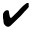	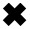	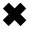
chemical bonding	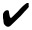	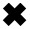	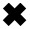
van der Waals forces	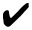	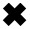	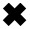
capillary forces	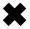	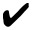	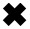
viscous forces	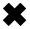	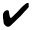	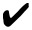

Adhesion that occurs under immersed conditions is greatly complicated by the difficulty in displacing water from the contacting interfaces and the ability of water to weaken many forms of bonds [40]. The relevant ones are described in the following.

#### van der Waals forces and chemical bonding

van der Waals forces are the sum of attractive forces between molecules that have regions of slightly negative and slightly positive charges. These forces are only effective over a very small distance, less than one nanometer [41]. Therefore, these forces are considerably weakened in the presence of water, which tends to form a separating film between the surfaces. For example on polyvinylsiloxane (PVS) surfaces, van der Waals forces are decreased to 12% of the value in air when submerged under water [42].

#### Capillary forces

Capillary adhesion occurs when a water film separates two hydrophilic surfaces in air. Pulling the surfaces apart will create a larger air–water boundary surface area. The surface tension of the liquid will resist to this increase and this is manifest as an adhesive force. According to [3] Laplace's law ought to be applied:

[7]
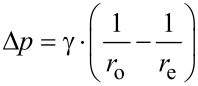


**Figure 4:**
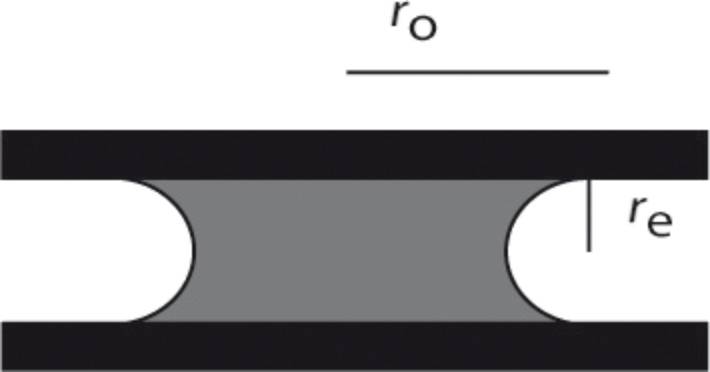
Two hydrophilic surfaces separated by water. Graph shows the location of the overall radius (*r*_o_) of the wetted surface and the radius of the curved edge (*r*_e_). The latter is half the thickness of the liquid.

The pressure difference (Δ*p*) can be calculated from surface tension (γ), the overall radius of the liquid (*r*_o_) and the radius of the curved edge (*r*_e_) (Figure 4).

In contrast, under fully immersed conditions the surface tension should be zero, so that generally no capillary forces will occur under these conditions. This is an important difference between adhesion in terrestrial and aquatic systems. Nevertheless, there are exceptions. Some terrestrial animals can step in droplets, e.g., on plant surfaces or even be completely submerged under water for a short time due to heavy rainfall. For example, the beetle *Gastrophysa viridula* can walk under water [43]. This beetle develops higher adhesive forces on hydrophobic surfaces compared to hydrophilic ones. The hydrophobic setose pads of the beetle hold air under water, so if it encounters hydrophobic surfaces the contact interface gets de-wetted, but not on hydrophilic surfaces. Additional capillary bridges between foot and substrate are formed due to the fluid secreted by the beetle and shear adhesion forces are in the same range as in air [43]. Similar results were described for geckos attaching to hydrophilic surfaces underwater and in air while no significant decrease in attachment forces were measured on hydrophobic surfaces under dry and wet conditions [29].

#### Viscous forces and Stefan adhesion

In the late 1800’s Stefan proposed a closed form solution to the problem of separating two rigid plates in a fluid [44]. If the plates are pulled away from each other in vertical direction, the fluid has to move inwards. Stefan’s solution was expressed in terms of the time it would take to separate the plates to a particular distance given an applied force. Recasting this equation into the force domain shows that force is proportional to the fourth power of the radius of the plates, the viscosity of the fluid, the speed of separation and the inverse of the separation distance. The equation for Stefan adhesion predicts very high forces for materials with the viscosity of mucus, but assumes rigid plates, and attempts to quantify the effect in limpets and tree frogs have yielded equivocal results [45–46]. It is clear that viscous forces play a role in the wet adhesion of tree frogs [46–47], and may also be important for some slugs or snails [3] or torrent frogs [30]. The application of Stefan’s equation is not unproblematic for biological cases though, because it ignores the material properties of the surfaces and is limited to unrealistic shapes. Smooth, and rigid plates are not what we usually find in nature, and elastic and structured surfaces of different shape will show a different behaviour. Moreover, other effects can cause failure sooner than predicted by Stefan’s equation. Under increasing stress, air bubbles in the viscous fluid can cause cavitation, which means that growing bubbles provide extra volume required for the plate separation [48]. Another process that causes instabilities is called air fingering, air moves in from the edge of the sample to the centre and by this brings the atmospheric pressure well into the sample [48]. These effects will be most important if the surrounding fluid is air. If the surrounding fluid is water instead, these effects are assumed to be different due to the incompressibility of water. Peeling is another component that can considerably reduce detachment force if the plates are not separated at right angle.

The low viscosity of air means that in terrestrial environments, it is hard to imagine an important contribution of viscosity. However, it is potentially very important where there is a secreted layer of fluid between the attachment organ and the surface in terrestrial systems. In aquatic systems viscous forces are obviously important due to the much higher viscosity of water compared to air. Moreover, the viscosity of the interstitial fluid might not be that of water because it is possible that some secreted mucus dictates the sliding force. The topic is further complicated by the fact that animals can secrete more than one material. Moreover, as the viscosity of the material increases there is a transition to glues. In particular, adhesive gels of gastropods can contain specific glue proteins with gel stiffening properties [49].

Determining the effects of compliant materials, irregular and divided surfaces, and non-convex surfaces should be a high priority for understanding aquatic adhesion. There has been considerable effort in the engineering world with regard to this area. In particular, the problems faced by tires on roads of varying roughness and wetness has driven the development of theories that may be applicable to biological systems [50–51]. These theories are difficult to assess in a biological context, but an examination of the parameters, surface roughness, substrate compliance, friction, and fluid properties, implies they will be useful. A further complication in water bears mention: The biofilm of fouled surfaces has a high effective viscosity and is also viscoelastic [26]. It is likely that an empirical understanding of viscous adhesion of animals to biofilms will have to be developed.

#### Glue

Many organisms use adhesive polymers to glue themselves to a substrate. Biological adhesives can vary widely in structure and capability, be remarkably complex and involve a large range of interactions and components with different functions [40]. The strength of the adhesive bond is determined by the biochemical nature of the adhesive secretion [52]. Many adhesives are non-specific and can adhere to many different types of substrates. Many of these glues form strong attachments under water, a process that is complicated by the difficulty in replacing water from the adhesive interface [40].

It is clear that the physico-chemical conditions are different under water than in air and therefore different kinds of glues are required. However, due to the complexity of the topic it is not possible to discuss these complex chemical issues in this review. Nevertheless, glue is probably the most common attachment mechanism in benthic marine animals, where the organisms are often exposed to strong currents in varying directions. Adhesives are used for long term fixation to the substrate, e.g., by mussels and barnacles [40], and glue is also used for temporary adhesion, e.g., in snails, flatworms and seastars [52–53]. For example, echinoderms and flatworms use a duo-gland system for attachment and detachment [16,54].

**Figure 5:**
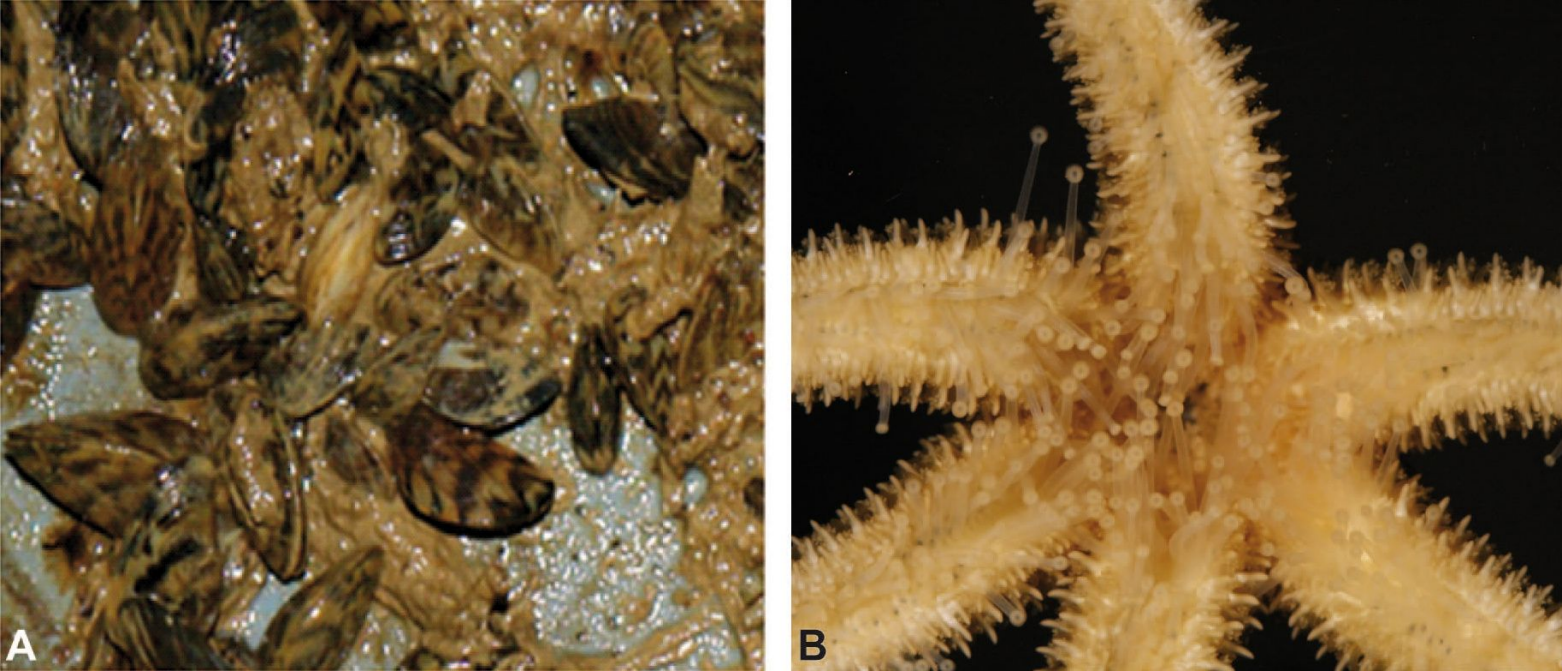
A) Zebra mussles attaching by byssal threads to a substrate, B) echinoderm foot (images: Petra Ditsche).

Many freshwater animals, such as molluscs and insect larvae, also use glue to attach to the substrate, often in running waters. For example black fly larvae place hooks in their secretion [22]. They are also able to secrete a sticky thread that helps them to reattach themselves again if swept away [55]. Some cased caddis larvae anchor their cases more or less permanently to the substrate with silk [56]. The invasive zebra mussel has become abundant on rocks and man-made structures in rivers and lakes in part because of its strong attachment by byssal threads [56–58] (Figure 5A).

### Friction

Friction, the resistance developed when one surface is moved across another, is complex and we will touch only on the main principles here. Friction does not depend on the sliding speed, but in general, kinetic friction (friction during movement) is lower than static friction (friction until movement starts) [19]. Moreover, friction is independent of the nominal (apparent) contact area defined by the geometry of the body. Instead, the real contact area between the two surfaces matters. The real contact area is defined by topography, material properties and normal load. Normal load is the sum of external load and adhesion [59]. There is a close relationship between friction and adhesion, and solids with a high frictional coefficient usually have stronger adhesive properties [38].

In terrestrial systems, we can distinguish between two cases in which friction plays different roles in adhesion, namely dry and wet. Dry friction occurs between dry, clean surfaces in a very dry atmosphere. In this case, the friction force is usually proportional to the real contact area, as it is actually the force needed to shear the junctions formed between the surfaces in contact.

In contrast, in wet friction a film of water or another liquid is involved. This liquid can originate from humidity in the air or from secretion by the animal. Under such boundary lubrication conditions there is a constant contact between the surfaces and friction is defined by physico-chemical properties of the bonds formed between the molecules of the fluid and the solid surface. This includes capillary forces, which can contribute considerably if the liquid has a high surface tension like water.

If the surfaces are fully immersed in water, we have a case of full-film lubrication. The surfaces are completely separated by the fluid and friction is defined by the nominal surface area and the viscosity of the fluid that has to be sheared. Moreover, in this case the surface tension is zero and no capillary forces contribute to friction. Thus, friction under submerged conditions will usually be reduced compared to wet friction. Complicating matters, the animal may secrete a fluid or material that modifies the properties of water to either increase or decrease friction in this full-film regime. The specific impact on friction will depend on properties of the secretion, such as surface energy and viscosity. Some monolayer films separating two surfaces can also decrease friction dramatically when the surfaces are immersed under water [60]. Under certain conditions such as an insect stepping into a water drop or a water spider walking under water, mixed lubrication might occur. In this case, friction is a mixture of cases of full-film lubrication and boundary lubrication.

**Figure 6:**
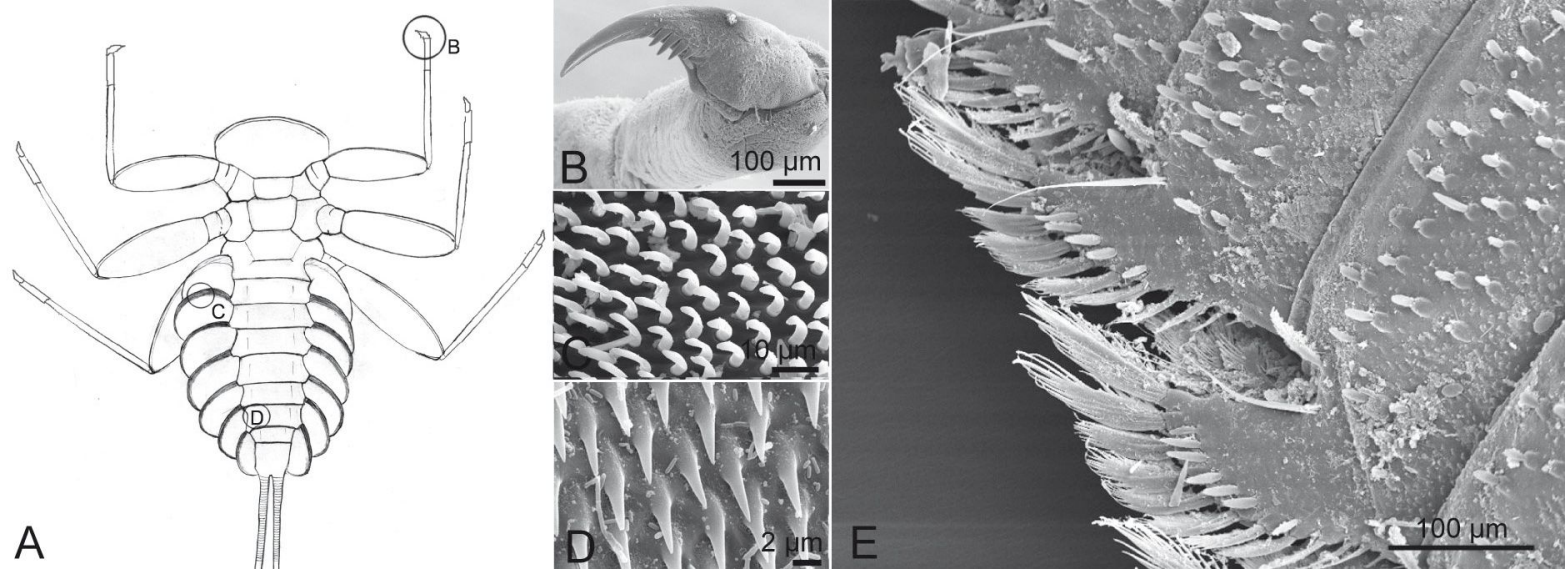
(A–D) Attachment devices of *E. assimilis* larvae: (A) ventral view, (B) claw of the foreleg, (C) setae of the pads on the ventral side of the gill lamellae, (D) areas with spiky acanthae on the lateral parts of the abdominal sternits. Reproduced with permission from [26]; (E) Structure on the distal edge of the ventral side of the beetle larva *Elmis s*p. Reproduced from [18].

The growth of biofilms and fouling organisms on aquatic substrates can have a significant impact on friction. The decreased tenacity of Northern clingfish on fouled surfaces is explained by the lubricating effects of the slimy parts of the biofilm decreasing friction at the margins of the suction disc [27]. Friction is often combined with other attachment mechanisms, such as the just mentioned suction. Friction also contributes to mechanical attachment mechanisms such as hooks, clamps and spacers. Thorns and other protuberances found on the underside of many torrential insects can also increase friction with the substrate as well as an increased surface area of the animal contacting the substrate [56]. Specialized friction pads, which increase friction on smooth and most rough surfaces, can be found on the underside of the gill lamellae of some mayfly larvae [61] (Figure 6) and in torrent dwelling fishes.

### Suction

The term sucker has been used for many attachment devices, so we want to clarify that we are just covering structures for which attachment is due to a difference between the pressure under the suction cup and the ambient pressure. In some cases this pressure difference is not the only factor in total attachment, as in cephalopods with hook-lined sucker disks.

**Figure 7:**
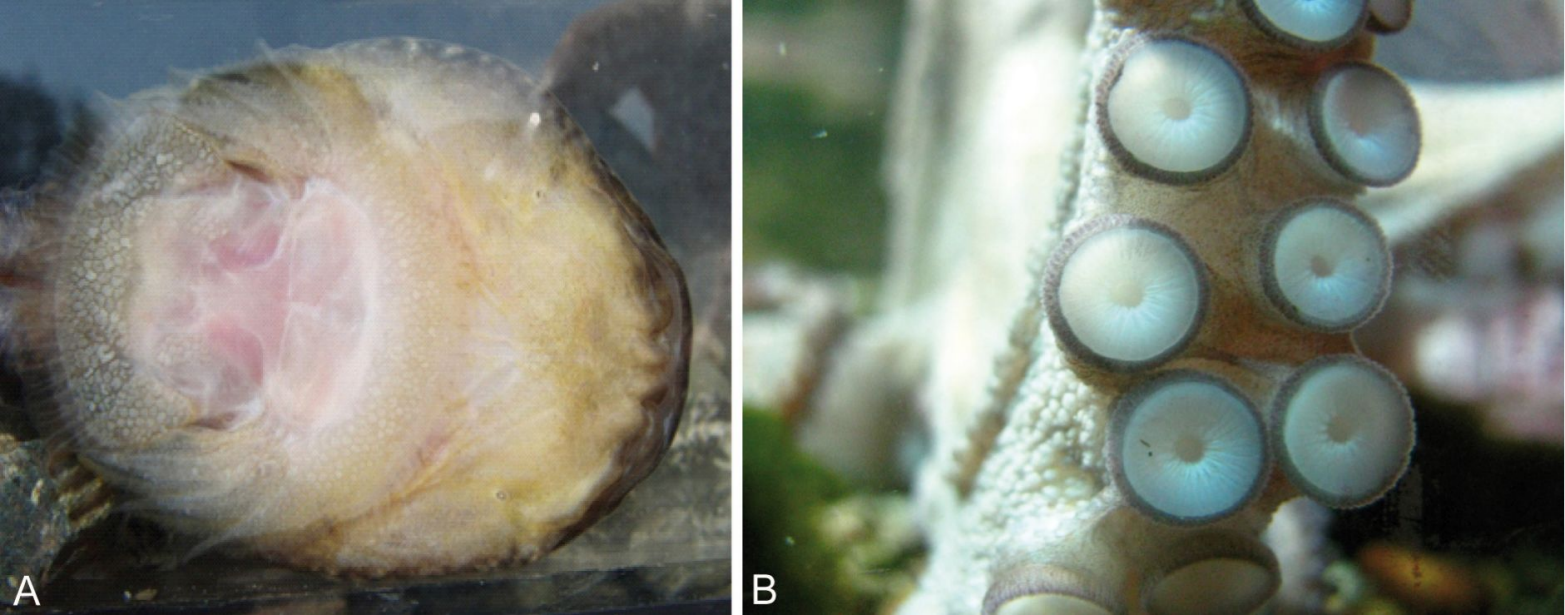
(A) Suction disc on the ventral side of Northern clingfish. Reproduced with permission from [27]. (B) Multiple suction discs on an octopus arm (image: Francesca Tramacere).

Suckers are common in aquatic animals. In freshwater we can find true suckers, for example in some fish, leeches and Blepharicerid larvae. The latter live in torrential mountain streams and can resist very high currents (*Hapalothrix lugubris* withstand flow velocities of 4.5 m/s) and generate attachment forces up to 8.4 g per sucker, which equals about 84 mN [36]. Gobiies, balitorid loaches and loricariid catfishes also have specialized suction discs, which help them to stay in place in the high-speed currents in stream environments [62–64]. Some gobies are even able to climb waterfalls by using a pelvic fin derived suction disc [63,65]. Moreover, lampreys are able to climb waterfalls with an oral sucker [66]. In marine systems, for example octopus, limpets and several lineages of fish have suction cups [67–69]. Clingfish (Figure 7), shark suckers, snailfish and lumpsuckers are marine taxa with a dedicated suctorial disc [37,70–71].

**Figure 8:**
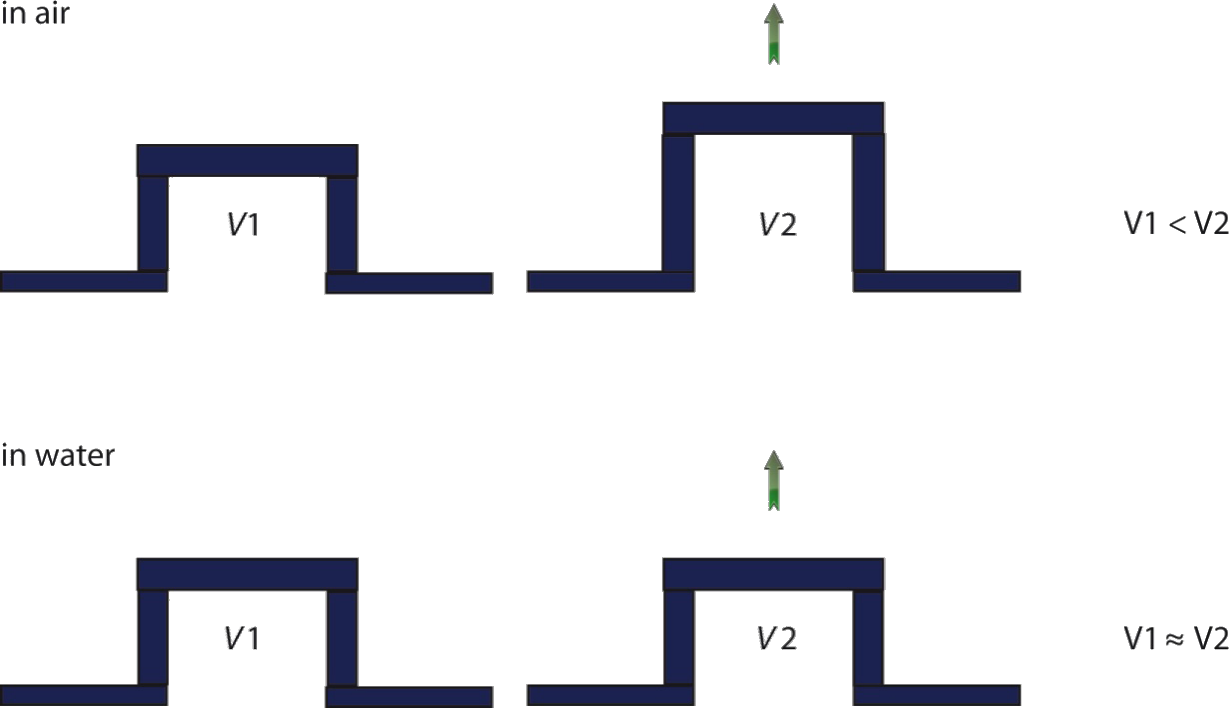
In air the volume of the cavity of suction cups increases. Due to the incompressibility of water the volume of suction cups stays the same while working under water.

In comparison to suction cups working in air, one of the most important differences is the incompressibility of water. Therefore, the volume of the water filled cavity will not change measurably (Figure 8). While pressures lower than a vacuum are not possible in air, this is possible if the suction cup is filled with water. For a vacuum (0 MPa) the tenacity developed at a maximal pressure difference would be 0.1 MPa at sea level. However, the pressure measured under an octopus sucker went negative in 35% of the cases and the lowest pressure measured was 0.168 MPa [72]. The same study shows that seawater can sustain negative pressure, but the values are not as low as for pure water. Particles and microbubbles in the seawater provide nucleating sites that stabilize the growth of larger gas bubbles [72]. Suction also depends on the surface energy of the substrate, as no negative pressures were found on non-wettable surfaces [68,72]. Many superhydrophobic surfaces are known for their ability to hold an air film under water for a varying time span [73–75]. Therefore, these surfaces could hold micro bubbles that serve as cavitation nucleating sites as in seawater. Whether this effect would occur after a long-time exposure of the substrates or at higher pressure has not been resolved. Ambient pressure also has an impact on cavitation [76]. It is possible that extremely high pressures can reduce the cavitation threshold, but this is just likely to matter at great depth.

The attachment force of the sucker (*F*_s_) is determined by the pressure difference (Δ*P*) and the area (*A*) of the suction cup:

[8]
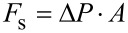


**Figure 9:**
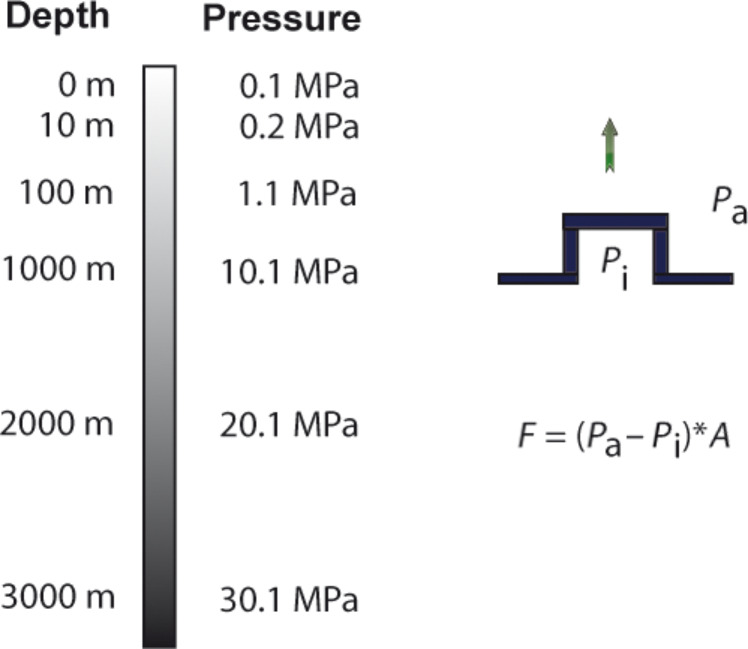
The pressure difference of the cavity under the suction cup and its environment determines the maximal possible suction force. As the pressure increases with water depth the theoretically maximal possible suction force increases with water depth as well.

For animals living far below the water surface the increasing pressure will also cause a different situation. As the hydrostatic pressure increases 0.100 MPa per 10 m depth, the possible pressure difference between the lower side of the suction cup and its environment increases. Thus, the theoretically possible attachment force increases as well simply by living in deeper aquatic regions (Figure 9). However, other factors such as limits of the muscles creating the pressure difference under the suction cup, and the ability to seal the edges of the suction disc will also influence the pressure difference. This explains why limpets show some effect of pressure on attachment but far less than theory would predict [35]. In contrast, the very strong suckers of some decapods are able to take advantage of the high pressure difference in deeper aquatic regions and pressure differences up to 0.83 MPa were measured. Such suckers are limited by cavitation of seawater at sea level, tenacity will increase with depth until a limit determined through morphology is reached [76]. The disc margin is often adorned with hairy structures, extremely soft tissue or secretions that serve to fix the edges of the disc as a dislodging force is applied [2,36,69].

Suckers typically work well on smooth substrates [19,56,77] because a seal between the disc margin and the substrate is more difficult to achieve on a rough surface. However, in some aquatic systems suckers develop higher tenacities on rough surfaces than on smooth ones [35,37]. The size of the suction disc has also a significant impact on the maximum roughness of the substrate to which it can attach [27].

### Mechanical principles of fixation: hook, lock, clamp and spacer

Though in broad strokes there should be little difference in the performance of these mechanical means of attachment in the two environments discussed here, there are some subtle but potentially powerful effects that should be considered. Since friction between the contacting surfaces contributes to the attachment forces, there could be quite different forces when the entire system is submerged simply because of the effect of a water film on friction. And, related to attachment force, it might take substantially more force to form an attachment in the aquatic environment because the viscosity and density of water make it harder to bring surfaces into close apposition.

**Figure 10:**
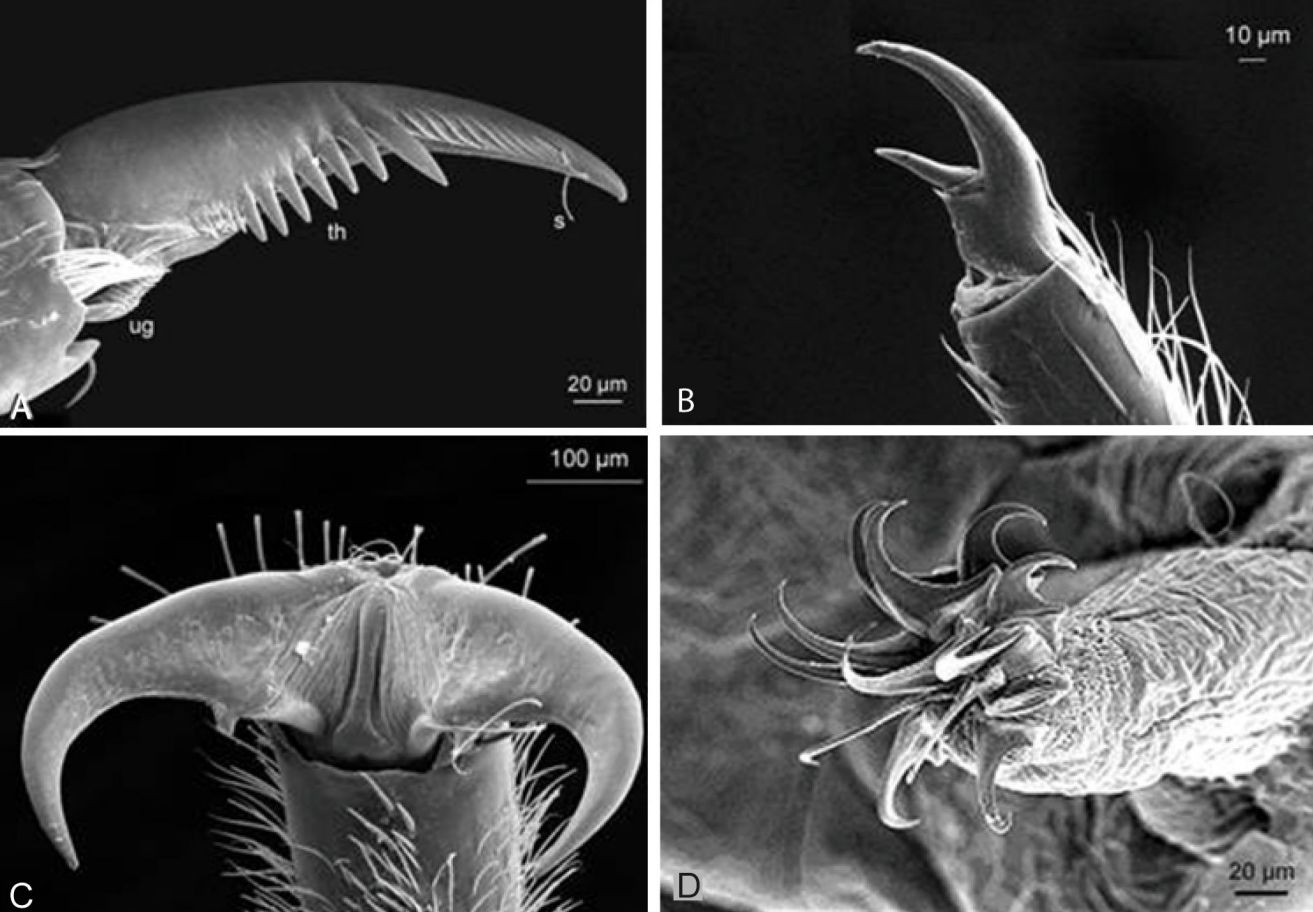
A) Tarsal claw of the mayfly larva *Baetis vardarensis.* Abreviations: ug unguitractor, th theeth, s sensilla. B) Tarsal claw of the second leg of caddies larva from *Rhyacophila sp*. dorsalis group, C) Double claws of the stream stonefly larva *Perla marginata*, D) Circlet of hooks on the proleg of a Chironomidae larva. Reproduced from [18].

Most arthropods living in flowing water have well-developed tarsal claws, with which they hold onto rough surfaces [56]. These claws show a variety of different shapes and sizes (Figure 10) and are the most common attachment devices of aquatic macroinvertebrates in both running and still water [78]. The larvae of some taxa, such as mayflies and caddis larvae usually bear one claw at their tarsi, while many others like stoneflies or several aquatic beetles have two tarsal claws (Figure 10). Double claws might act in the same direction or in accordance to the clamp principle (or something intermediate). Free-living caddis larvae like *Rhyacophila* have additional claws like grapples on their posterior prolegs. Circlets of outwardly directed hooks imply the spacer principle. They occur on the prolegs in larvae of several Diptera taxa such as Chironomidae, Diamesinae, Simuliidae and Deuterophlebiidae [79]. While the hook circlets of the Simuliidae are only engaged in secreted silk mats, those of the Deuterophlebiidae are used directly on the stones. In contrast to the clamp mechanism, in the spacer principle the hooks press outwardly directed. The lock principle is not very suitable for attachment to substrates as it needs two specialized surfaces, but very common for the connection of body parts or during copulation [2]. In marine environment claws are found in many arthropods.

While the immersion itself does not show considerable effect on mechanical interlocking principles, the secondary effect of the fouling of aquatic surfaces can cause a significant impact on attachment. On smooth substrates a biofilm cover can even increase friction forces of claws [26]. The claws can pierce the gel like part of the biofilm and interlock inside with attached organisms of the biofilm. Moreover, the higher viscosity of the biofilm gives additional resistance. In contrast, on rough substrates the opposite effect takes place [26]. Here the lubricating effect of the gel like part of the biofilm that separates the substrate and the claw dominates.

## Conclusion

Attachment in the aquatic realm is not just quantitatively different from that on land, but it is qualitatively different. Completely different phenomena are responsible for the most significant dislodgement and attachment forces under each of the two conditions. The constraints on attachment, and the opportunities offered by secure attachment, are different in the two realms and will require very different approaches to systematizing and characterizing biological diversity with respect to this parameter. Many open questions remain with regard to aquatic attachment, and some can be approached by comparing the more thoroughly studied terrestrial environment.

A major impediment to systematizing attachment performance across environments is the lack of standardization of methods and the difficulty of scaling up small experimental units to the performance of the whole animal. We suggest that as research in this area progresses it would be very useful to make raw data accessible in open source outlets so that derived performance parameters can be computed ex post facto as theoretical models and technology improve. It is particularly important to broaden the knowledge base about the performance of animals as a whole, because this is what is evolutionarily selected over time and what will lead to the most immediate ecological insights.

Size effects on attachment, especially in the submerged case, might provide key insights into selective pressures and may yield clarity regarding the ecology of the organism. Paying attention to the scaling of detachment forces, for example, leads to some questions worth pursuing. Gravity and inertia dominate the dry world while lift and drag rule the water currents. Forces due to gravity scale with mass and, therefore, also with the cube of length, while those from lift and drag are scaling with the square of length. Do aquatic organisms have no size constraints because the effects of gravity are ruled out by lift and drag? Are the biggest attached organisms aquatic? Indeed, some benthic animals such as the giant sea star *Pisaster giganteus* can become very large. However, the topic is complicated by other factors influencing the magnitude of the detachment forces. First, in wave-swept environments, the acceleration reaction force can be high and as it depends on the volume, it scales with the cube of length as well. This is assumed to be one reason why organisms inhabiting the marine intertidal region are often small compared to the ones living in the subtidal region, where waves are less pronounced [80]. Second, if benthic animals are small enough to reside in the boundary layer, which develops over the surface of substrates, they might take advantage of the reduced flow velocity close to the substrate. Thus, being very small might be of advantage under certain circumstances, for instance in high-flow-rate streams [81–83]. Actually, most macrozoobenthos organisms in streams are very small, even considerably smaller than the macrozobenthos of the marine intertidal. Third, flow forces acting on an animal depend on the flow velocity, which can show huge variations between different habitats and even inside the same habitat. Fourth, the shape and other properties (such as elasticity) of the animal also strongly influences drag and lift forces. Even more complicated, their effect on the different flow forces can vary. For example, a shape that reduces drag does not necessarily reduce lift and vice versa. Moreover, the behavior and way of living of an animal strongly influence the detachment forces acting on it. Beside mechanical factors also biological factors can limit the size of organisms [84].

Another interesting point regarding size is the relation of the animal size to the maximal possible attachment forces. Our calculation showed that for the same hypothetical animal much higher detachment forces could occur in aquatic systems at high flow velocities (Table 3). Therefore, the question arises, whether the highest attachment forces in relation to the body size occur in aquatic systems. To test this idea we need a more complete picture of size parameters than is often reported.

A most intriguing aspect of this review is the demonstration that we need better understanding of viscosity-mediated attachment. Viscous adhesion is clearly important to the real, biologically messy, and sometimes mucus-laden real world, but the underlying processes are not fully understood for submerged cases. The community needs useful theoretical models that account for the compliance of the substrate and the attachment organ, the surface energies and the shapes of real world examples of attachment.

Submerged organisms must cope with water and also with ubiquitous biofilms. The complexities of a pure liquid pale in comparison to the presumably non-Newtonian behaviour of this viscoelastic polymer, adherent to virtually every submerged surface. The effect must depend on the type of the attachment device, and also with the thickness and composition of biofilms. This complicates real world performance measures, but the issue must be faced sooner rather than later if we are to put attachment in an ecological context in the aquatic world. Early investigations in this area demonstrate the complexity of the interaction between attachment organ and substrate, with examples of increases and decreases in tenacity with biofilm growths.

We are working in an age of unmatched technology for imaging and experimentation, with a rapidly diminishing core of natural historians [85]. The investigation of aquatic adhesion is going to be successful as disparate fields examine the same problems, and first hand observation of nature becomes more valuable not less. This review should serve as a call not only for better theory or experimentation, but for a more complete, thorough and detailed observation of the attachment phenomenon in a natural setting.
